# Forest therapy using virtual reality in the older population: a systematic review

**DOI:** 10.3389/fpsyg.2023.1323758

**Published:** 2024-01-17

**Authors:** Davide Clemente, Luciano Romano, Elena Zamboni, Giuseppe Carrus, Angelo Panno

**Affiliations:** ^1^Experimental and Applied Psychology Laboratory, Department of Human Sciences, European University of Rome, Rome, Italy; ^2^Department of Education Science, Roma Tre University, Rome, Lazio, Italy

**Keywords:** virtual reality, forest therapy, virtual natural environment, virtual forest environment, exposure to nature, older people, natural environment, virtual environment

## Abstract

**Introduction:**

As life expectancy increases, more attention needs to be paid to their mental and physical condition. Many older patients are also bedridden, which makes some treatments, like *in vivo* exposure to natural environments, more difficult to be applied. This study aimed to systematically review articles that include interventions combining virtual reality and forest environment, targeting a sample of older people.

**Methods:**

Based on PRISMA guidelines, we conducted a literature search in three databases (EBSCO, PubMed, and Scopus), plus gray literature (OpenGrey). We considered only studies that used forest settings via virtual reality and included a sample with age ≥ 65.

**Results:**

After the screening and eligibility stages, 7 articles have been included.

**Discussion:**

The study underlines the need to implement research in this direction to standardize effective procedures that can be used to improve the mental and physical health of the older people and caregivers, while also reducing social costs.

## Introduction

The older people represent the most fragile group of the population, toward which, at times, little attention is paid. The World Health Organization (WHO) defines the age of 65 as the transition condition to the “older” (Kowal and Dowd, 2001), and it is confirmed by some studies (e.g., Crews and Zavotka, 2006). Most Western regions show a trend toward an aging population (e.g., Cuijpers et al., 2006; Arean et al., 2008). As a result, more and more people are developing chronic diseases, which in recent years, have seen significant improvements in their better management (Uijen and van de Lisdonk, 2008; Murray et al., 2015). This leads to a significant reduction in mobility, consequently increasing the fragility of physical structures, leading these individuals to live increasingly sedentary lives (El-Khoury et al., 2015). In this regard, the academic literature has shown a strong correlation between chronic diseases and sedentary lives (e.g., Vancampfort et al., 2017). To ward off the adverse effects of old age, various interventions have been tried out to reduce sedentariness and increase psycho-physical well-being (Sollami et al., 2017; Buedo-Guirado et al., 2020). These include forest therapy, which has also been tested in recent years (e.g., Sung et al., 2012). Forest therapy consists of all activities involving the forest environment (e.g., exposure to the forest environment, walking in the forest, etc.) for the promotion of health and well-being (Kotte et al., 2019; Zhang et al., 2020). From a medical point of view, Forest Therapy appears to have a preventive role on chronic diseases and a protective role on health, leading to restorative outcomes (Mattila et al., 2020). Moreover, several studies have revealed the pivotal effects of forest therapy in reducing stress (Jung et al., 2015), decreasing cortisol levels (Sung et al., 2012), as well as in strengthening the immune system (e.g., Li and Kawada, 2011; Chae et al., 2021).

Research has shown how Forest Therapy enables people to control stress, anxiety and depression levels and generates effects primarily on the immune system, respiratory and cardiovascular systems (Song et al., 2016). An additional practice used is Shinrin-yoku, which is used as a synonym of forest therapy or as a part of forest therapy (Rajoo et al., 2020), a technique involving bathing in the forest through total immersion engendering well-being as well as benefits for physical health (Reese et al., 2022). Like Forest Therapy, such an intervention reduces depression, anxiety and heart rate, increasing the perceived positive affect (Hansen et al., 2017). The limitation of this intervention is the exposure to a natural setting *in vivo*. This feature might be inapplicable to some sub-populations of older people, as they may be bedridden or have walking difficulties. A viable alternative to *in vivo* stimulation has been, for years now, virtual stimulation via virtual reality (VR) (Spano et al., 2023). VR is defined as a technology that uses special equipment, such as head-mounted displays, to reproduce simulated environments, allowing individuals to undergo an immersive experience (Yu et al., 2018). The use of VR has also been applied in interventions with older people in order to reduce the isolation and monotony of an overly sedentary life due to medical conditions (Baker et al., 2020). This combination allows interaction with virtual environments by creating a realistic sense of presence within the computer-generated environment (e.g., Stanney and Zyda, 2002). In this way, despite the physical hindrances, depressed mood and isolation of the older people can be reduced, and accordingly producing benefits for the medical condition, as well (Woodford and Fisher, 2019). Nonetheless, despite few studies that have combined virtual reality technology with the principles of forest therapy (Annerstedt et al., 2013; Li et al., 2020; Mattila et al., 2020; Yu and Hsieh, 2020), these still appear to be poorly oriented to an older people sample. Therefore, the present review aims to explore the benefits of VR based interventions with forest environments on older people by systematically summarizing the scientific literature on the topic (Table 1).

**Table 1:** PICOS of the systematic review.

P	Old people (definition by WHO ≥65)
I	Use of virtual reality with images/videos of forest environments
C	Comparison of using virtual nature with non-virtual landscapes, cityscapes, real nature landscape conditions, or other stimuli (e.g., sound-related stimuli) VR combined with exercise or movement
O	Improvement of physical and/or psychological health
S	Experimental studies/randomized controlled trial

## Methods

For the present systematic review, the guidelines of the Preferred Reporting Items for Systematic Reviews and Meta-Analysis were followed (PRISMA).^1^

### Search, screening, and selection strategies

The following inclusion criteria were adopted: (a) experimental studies or RCTs (randomized controlled trials); (b) studies with older people samples (age over 64 years, consistent with the age that the World Health Organization defines to indicate the transition to older people status) (Kowal and Dowd, 2001); (c) studies that implemented a VR intervention and used a forest environment. The following exclusion criteria were adopted: (a) nonexperimental studies, (b) studies having a sample age of less than 65 years, (c) studies with interventions other than VR, (d) studies with VR interventions, but without forest environments. The research, therefore, took the PICOS “shown in.

The review was conducted through three databases: EBSCO, PubMed and Scopus. The following string of keywords, using Boolean operators (AND and OR), was adopted: *“elderly” OR “aged” OR “older” OR “geriatric” AND “virtual reality” OR “vr” AND “forest” OR “forest therapy” OR “nature therapy” OR “virtual nature” OR “forest bathing” OR “shinrin-yoku.”*

A total of 97 articles were identified, 11 of which were duplicates and were therefore eliminated. Of the initial 86 articles, following an initial selection, 80 were excluded for the exclusion criteria explained above and because off topic. The gray literature was also investigated through the OpenGrey database, but the search yielded no results.

In filter selection, filters were applied by sample age (65 years and older) and VR use with forest environments. The search, conducted through 3 databases, was completed on February 16, 2023. EBSCO produced 12 results, 2 of which were evaluated as eligible (Moyle et al., 2018; Yuan et al., 2022). The PubMed search yielded 53 articles, but none were evaluated as eligible because they were not consistent with the inclusion criteria.

**Figure 1:**
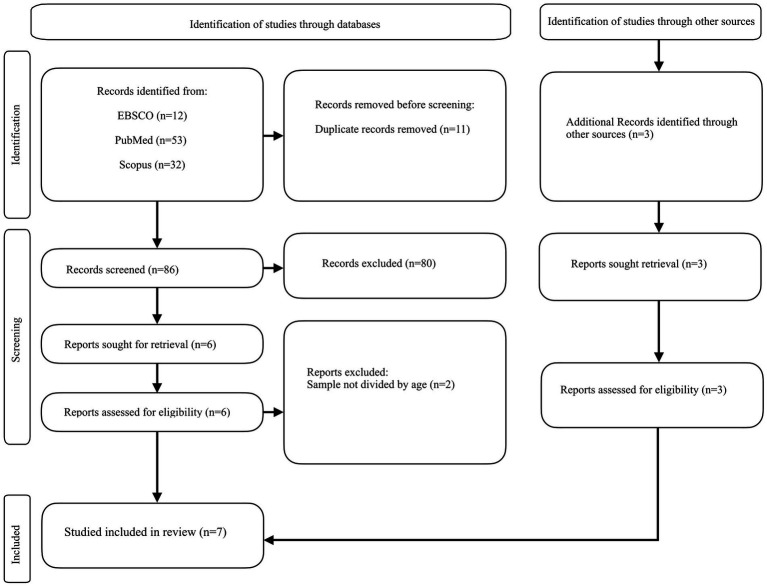
Flowchart of the process of initial literature search and extraction of studies meeting the inclusion criteria.

The search through Scopus identified 32 articles, of which 4 articles were considered, but 2 of these were excluded because although it included a sample of older people within it, this was not divided by a sample with other ages considered, and the results were not divided by age. Therefore 2 articles were considered eligible (Graf et al., 2020; Appel et al., 2022). Research was also conducted through other sources such as bibliographic citations (Appel et al., 2020; Brimelow et al., 2020) of study (Appel et al., 2022), and website (this paper is present also in Scopus, but by subsequent and further research, this paper was also selected as the authors noted its eligibility Lundstedt et al., 2021). The selection of articles was carried out independently by two of the authors (Authors 1 and Authors 3); there were no instances of disagreement between the two authors. In case of a non-univocal decision on an article, the opinion of Authors 2 would be sought. All information about the literature search process and study extraction is shown in the flowchart shown in Figure 1. Characteristics of the studies shown in Table 2.

**Table 2:** Characteristics of the studies.

Author(s)	Year	Country	N. studies	Aims and/or RQ	Study design	Sample size	Participants characteristics	Outcome measures	Tools	Type of VR stimulation	Main results
Yuan et al.	2022	China	1	Taking a break of a few minutes in a VR forest has significative effects on effect of elderly. Taking 3 short breaks in a VR forest over 3 consecutive days results in psychological improvements. Taking a break of a few minutes in a VR forest does not significantly decrease blood pressure. Taking 3 short breaks in a VR forest in 3 days decreases pressure levels of elderly individuals. Taking a break of a few minutes in a VR forest has more obvious psychological restorative effects. Taking 3 short breaks in a VR forest over 3 days results in a greater blood pressure reduction for introverted elderly individuals than for extroverted ones.	RCT (Randomized control trial)—two groups	63	(Mage = 81.98 years, SD = 7.15, 21 males) from an elderly care	The participants’ affect states were measured with the short version of the PANAS scale. Stress recovery was measured with ROS scale adapted. SBP and DBP were measured with YE630AR, YUYUE, China. Introverted/extroverted personalities: two-item 5-point scale Intention to undertake real forest therapy was measured by some questions. Pain scale: faces and numerical pain scale were used to assess participant’s pain before and after each chemotherapy. Distress screening tool: The Houston Methodist Cancer Service’s Distress Screening Assessment Tool. Saliva cortisol testing for stress	VR SHINECON AIO5 Oculus Quest	Forest environment: Black Valley National Forest Park, Chongqing, China, which is famous for its primeval forest and vegetation. A mix of ambient sounds, such as birds singing and running water, was also part of the experience. Nature Treks software	H1: Taking a short break could significantly increase the positive affect and decrease the negative one. H2: 3 short breaks in a VR forest over3 consecutive days can lead to continuous psychological improvements. H3: Taking a break of a few minutes in a VR forest does not significantly decrease elderly. H4: 3 VR forest experiences over 3 days on elderly people’s blood pressure did not have significant effects. H5: Taking a break of a few minutes in a VR forest produces restorative effects. H6: The interactions on SBP were not significant. H7: One way ANOVA showed that the VR group’s intention to receive real forest therapy was higher than those of control group
Lundstedt et al.	2021	Sweden	1	The purpose of the study was to acquire an understanding of how residents and staff at a residential care facility may use and experience different virtual natural environments, and thus aid the design of virtual natural environments.	Qualitative (i.e., collecting data through interviews and observations)	7	4 women and 3 men ranging from 67 to 91 in age with a Mage = 88. The number of years that the residents had lived at the residential care facility ranged from 1 to 6 with a mean value of 3.4. The study participants were diagnosed with various mental and/or physical health conditions, which, in most cases, were the initial reason behind moving to a care facility	Took notes of observations of the residents’ reactions as expressed through oral communication, and verbal and bodily expressions. Noted observations of the usability of the VNEs and the situation in general. Interviewed the staff members who had been the most frequently involved in the VR sessions regarding their views of the usefulness of such VR sessions at residential care facilities and their general impressions. The interview was audio-recorded and later transcribed. The staff was also asked to respond to questionnaires regarding the participating residents’ gender age, number of years, residing at the facility, and diagnosis/reason for taking residence at the facility.	OculusGo–Samsung Odyssey –HTC Vive	360° blue space videos on Oculus Go -theBlu on Samsung Odyssey-HTC Vive Vr Island	Emotional reactions: The participants expressed emotions during the VR coffee sessions. Aesthetical pleasure: Most expressions of aesthetic pleasure were in relation to VR Island. Joy: participants express joy through verbalizations and not. Fascination/mind-blowing realization: through body language and verbalization residents expressed a fascination for the VR technology (especially in theBlue), and the feeling of presence. Discomfort Expressed: residents experienced mild discomfort when driving through or too close to objects when they expressed the water in VR Island appeared cold and stormy. General positivity: other distinctive positive responses were expressed by the participants. Negative reactions: anger, boredom and dissatisfaction. Expression of interest: people showed more positive expressions than negative ones in VNEs. Usability matters: OculusGo HMD was considered flexible and smooth, the HTC Vive HMD was perceived heavy. Some participants had difficulties with VR technology in using the index finger button on the controller.
Appel et al.	2022	United States	1	Evaluate the feasibility and potential benefits of introducing VR therapy to veterans and residents in a long-term care health center, with the main goal of reducing responsive behaviors for individuals living with dementia	A prospective, longitudinal, open, non-randomized interventional clinical trial based on convenience sampling	33 veterans	(12 female) with an average age of 91.6 years (SD 5.9)	Quantitative and qualitative data were collected from two sources: (1) Electronic patient record (EPR): (demographic data and clinical data). Baseline measures were based on validated RAI-MDS. 2.0 instruments. (2) The data collection intervention (four sections): Open-ended questions related to pain and reactive behaviors; 5-point Likert scale completed by RTs to record their perceptions of the impact of VR on a resident during a session. Administration of the PAINAD (before sessions); open-ended questions related to acceptability, liking, and willingness to use VR again.	An Oculus Go VR HMD	360° VR movies that include various nature scenes. RTs assisted in the creation and selection of additional customized content for participants by filming 360° videos of areas of interest to participants. The research team provided guidance to RTs on how to create and select VR content (e.g., no first-person movement, minimal changes in sound and lighting) to reduce the possibility of simulator sickness.	In most sessions (61%, 68/111), no reactive behaviors were observed during or after VR. The reactive behaviors identified were classified as wandering, agitation, negative affect, a combination of wandering and negative effects, and related to the activity of packing one’s belongings. In 46% of the targeted sessions, participants did not exhibit the reactive behaviors usually triggered by the identified environmental event. No PRN medication was required or administered for pain during any of the VR therapy sessions. Participants in the targeted sessions had higher PAINAD scores on average (*M* = 1.54) than those in the scheduled sessions (*M* = 0.78) at the beginning of the session. In 63% of the sessions, participants found HMD VR comfortable.in one-third of the sessions, residents seemed to reminisce during and/or after VR therapy. Reminiscence was observed more frequently in scheduled sessions.
Appel et al.	2020	Canada	1	Establish whether it is feasible to use immersive VR technology as therapy for older adults who have reduced sensory, mobility and/or impaired cognition. This includes evaluation of tolerability, comfort, and ease of use of the HMD, and of the potential for immersive VR to provide enjoyment/relaxation and reduce anxiety and depressive symptoms.	Clinical trial	66	Older adults (mean age 80.5, SD = 10.5) with varying cognitive abilities (normal = 2 8, mild impairment = 17, moderate impairment = 12, severe impairment = 3, unknown cognitive score = 6), and/or physical impairment s, entered a multi-site non-randomized interventional study in Toronto, Canada	A modified version of the STAI. A modified version of the MiDAS questionnaire Cognitive status and scores on standardized tests of cognition (MMSE, MoCA, CPS) were obtained from chart reviews	Samsung Gear VR HMD	Five different scenes (45 s to 3 min each) were presented sequentially. This 6 min video was automatically replayed from the beginning once all five scenes were experienced. Scene 1 featured a rocky shore, and waves; Scene 2 featured an open field with various colored foliage blowing in a gentle autumn wind; Scene 3 featured a dense forest with tall pine trees swaying in the wind; Scene 4 featured a black stone beach and ice water waves surrounded by a tall glacier, Scene 5 an aquamarine beach with gently flowing waves, bright blue sky and a family with a child and dog in the distance	All participant completed the study with no negative side effects reported; the average time spent in VR was 8 and 76% of participants viewed the entire experience at least once. Participants tolerated the HMD very much and had positive feedback feeling more relaxed and adventurous. 76% wanted to try VR again. Better image quality was suggested to improve the experience
Graf et al.	2020	Germany	1	The paper looking for an answer to these two questions: Is the VR game “VR forest walk” a suitable medium for elderly people regarding usability, technology acceptance, and sense of presence? Is a VR game capable of enhancing elderly people’s moods?	A mixed-method design combining quantitative (standardized questionnaire es) and qualitative (observation n protocol interview) measures	14	Participants who were at least 65 years old and met the inclusion criteria were considered eligible. 8 females the age ranging from 66 to 84 (*M* = 76.8, SD = 5.78)	Igroup Presence Questionnaire (IPQ). The Technology Usage Inventory (TUI). The Positive and Negative Affect Schedule (PANAS). Game Experience Questionnaire (GEQ) The Flow short scale (FKS) Simulator Sickness Questionnaire (SSQ)	Oculus Go	Virtual Forest Walk—Bella the virtual dog—Cognitive Tasks (Mini-Games)	Mean SSQ values related simulator discomfort showed low mean values. The IPQ results show that participants perceived a high sense of presence. There was a significant difference between the subscale of technological anxiety before and after the VR experience After the VR experience, participants rated their anxiety toward VR technology on average about 3.71 points lower than before. Ease of use shows a high mean with a maximum score of 21. The results indicate an increase in positive affect values but the difference was not significant. The values of negative affection are very low overall but show a decreasing trend in negative feelings although not significantly. The GEQ recorded moderate average data in relation to immersion competence a positive affection and higher averages in relation to immersion and positive affection. In addition, the “VR Forest Walk” was described as neither challenging nor negative. Participants experienced high levels of $ow during the VR experience. Regarding the subscales worry, smooth progression and absorption, participants showed low worry scores during the VR experience and showed high scores for smooth progression and absorption.
Moyle et al.	2018	Australia	1	To measure and describe the effectiveness of a Virtual Reality Forest (VRF) on engagement, apathy, and mood states of people with dementia, and explore the experiences of staff, people with dementia and their families	A mixed-methods study (e.g., video recorded Observations and interviews)	10 residents +10 family members + 9 care staff	Mage of residents = 89 (4.97)	Observed Emotion Rating Scale (OERS) Person– Environment Apathy Rating (PEAR). Type of engagement. Each resident’s engagement during the Virtual Reality Forest experience was coded into 3 types: self-engagement: the resident engages in the activity without encouragement; (a) facilitated engagement: engagement in the activity is encouraged and supported by another person; and (c) no engagement: the resident is not engaging in the activity. The average duration of time spent in each type of engagement was used to describe to engagement of the resident.	A large interactive screen designed by Alzheimer’s Australia Vic (2016). The seasons and various animated objects can be manipulated through Microsoft Kinect^®^ motion sensors allowing participants to interact with the scene through hand and arm movements	The Virtual Reality Forest uses video game technology, involving vivid graphics and motion sensors, to create an interactive and immersive environment. The Imagery of the Virtual Reality Forest includes a river spanned by a bridge winding through trees and flowers, which is accompanied by a background soundtrack incorporating peaceful white noise and forest sounds, such as bird calls.	Residents with dementia expressed significantly more pleasure (*p* = 0.008) and vigilance (*p* < 0.001) during the virtual reality forest experience. Fifty percent of residents also expressed higher levels of anxiety/fear during the experience than previously established norms for people with dementia (*p* = 0.016). No anger or sadness was observed during the virtual reality forest experience (*p* > 0.05). An overall significant effect of environmental stimulation was found for time observation (*p* = 0.004), also indicating that the VR Forest had stimulating qualities. A significant effect of apathy was also found for the observation of weather (*p* = 0.02). The results of PEAR analyses suggest that participating residents were immersed in the Virtual Reality Forest experience, resulting in reduced apathy during the experience, but the effect was not maintained.
Birmelow et al.	2020	Australia	1	To determine the feasibility of using virtual reality (VR) for residents with and without dementia in the residential aged care (RAC) environment (also referred to as nursing homes or long-term care)	A mixed-methods study (Consisted of quantitative validated observer tools, a resident feedback survey, and staff interviews)	13	Residents were aged between 66 and 93 years with a mean of 82–8 years (9 female—4 male). The mean time residents had spent in the RACF was 21 months (Range: 3–58 months). Residents were excluded if they had symptoms or a diagnosis of contagious conditions, serious ill health, or were in palliative care.	Observed Emotion Rating Scale (OERS) Person–Environment Apathy Rating (PEAR) A series of structured questions at the end of their VR session. Residents were observed for signs of simulator sickness. Signs and symptoms of simulator sickness were defined by the item on the Simulator Sickness Questionnaire.	A Samsung Galaxy S7 (weight = 152 g), preloaded with an aged care VR library, in tandem with a Samsung Gear VR headset (weight = 345 g) was used to create a fully immersive VR experience for residents	The 360-degree contained within the library consisted of relaxing scenes, such as underwater themes, beaches, farmyard animals, travel destinations, and snowscapes that were specially created for the aged care industry.	The VR experience significantly reduced apathy in residents (Z = −2.818, *p* = 0.005). Total scores reduced from a mean (standard deviation) of 15. (−6.11) to 11.38 (3.93). This was because of observations of increased facial expression (*Z* = −2.489, *p* = 0.013), eye contact (*Z* = −2.070, *p* = 0.038), Physical engagement (*Z* = 2.887, *p* = 0.004), verbal tone (*Z* = −2.428, *p* = 0.015), and verbal expression (*Z* = −2.714, *p* = 0.007). VR did not significantly measures of the OERS; no significant increase in fear/anxiety was observed. A trend was observed for increased pleasure (*Z* = −1.725, *p* = 0.084) and general alertness (*Z* = −1.639, *p* = 0.101).

### Characteristics of the included studies

Seven articles were included in this review (Moyle et al., 2018; Appel et al., 2020; Brimelow et al., 2020; Graf et al., 2020; Lundstedt et al., 2021; Appel et al., 2022; Yuan et al., 2022). These articles were published from 2019 to 2022 and were conducted in the following countries: China (Yuan et al., 2022), Sweden (Lundstedt et al., 2021), Australia (Moyle et al., 2018; Brimelow et al., 2020), Canada (Appel et al., 2020), Germany (Graf et al., 2020), and United States (Appel et al., 2022). The number of participants enrolled ranged from a minimum of 7 to a maximum of 66. All studies had a sample population aged over 65 years. The highest mean age of participants was in the study of Appel et al. (2022) (*M* = 91.6), while the lowest was in Graf et al. (2020) (*M* = 76.8). Among the 7 selected studies, Yuan et al. (2022) conducted a Randomized Control Trial. Lundstedt et al. (2021) conducted a qualitative study. Furthermore, Appel et al. (2020) and Appel et al. (2022) conducted a prospective, longitudinal, open, non-randomized interventional clinical trial. Besides, Graf et al. (2020), Moyle et al. (2018), and Brimelow et al. (2020) carried out a mixed study. In detail, Graf et al. (2020) combined qualitative questions and quantitative measures; Moyle et al. (2018) alternated video recorders, observations, and interviews; Brimelow et al. (2020) combined validated quantitative observer instruments, a resident feedback survey, and staff interviews.

### Visors used

Different viewers and images to create virtual environments were adopted in the selected studies. Yuan et al. (2022) used VR SHINECON AIO5, a head-mounted display with gyroscopes, nine-axis sensors, headphones, and other accessories. Lundstedt et al. (2021), in their study, decided to use three different viewers. (1) An Oculus Go, a self-contained display without cables that can be strapped to the viewer’s head or held in hand and used as if it were binoculars. (2) the Samsung Odyssey, which is a head-mounted display that requires a cable connection to a computer. (3) the HTC Vive; this is an HMD that requires a cable connection to a computer. Instead, Appel et al. (2020) decided to use the Samsung Gear VR HMD (VR hardware system consisting of Samsung S7 smartphones to view VR videos), Samsung VR HMD with a viewing screen and limited real-world vision, Sennheiser HD 221 headphones to play the sound of the movies and minimize the sound of the surrounding environment, and VR technology replaceable health face pads for individual use. Like Graf et al. (2020) and Lundstedt et al. (2021) also used the Oculus Go. The Oculus Go device was also used in the study by Appel et al. (2022), they also used an HMD VR device. Unlike the other authors who used mobile devices, Moyle et al. (2018) used a large screen and provided patients with Microsoft Kinect^®^ motion sensors. In the study by Brimelow et al. (2020), the authors used Samsung Galaxy S7 devices as sensors with Gears VR visors.

### Images used

Yuan et al. (2022), in their study, used images related to the Black Valley National Forest Park in China, and environmental sounds, such as birdsong and running water, were also reproduced. Lundstedt et al. (2021), in their study, used 3 different conditions about natural environment. A Virtual Island with various environments (e.g., forest environments with trees, a beach, a meadow, etc.), a 360° video of blue space (with Oculus Go), shot at various coastal locations in Cornwall, England, and a virtual nature environment “theBlu” (interactive underwater environments in real time, with Samsung Odyssey). Appel et al. (2020) have chosen five scenes: a dense forest with tall pine trees swaying in the wind; a rocky shore and waves; an open field with colorful foliage blowing in an autumn wind; a black stone beach and waves of icy water surrounded by a tall glacier; a beach with gently flowing waves, a bright blue sky and a family with a child and a dog in the distance. In the study of Graf et al. (2020), the virtual environment used in this experiment, named “VR Forest Walk,” comprises a forest, a mountain landscape, and a virtual ocean. Also, they have used cognitive tasks (Animal Bingo and Memory Parkour). A virtual dog was also played in the virtual environment, whose purpose was to provide companionship to the subject involved, which is not thrilling or exciting. Appel et al. (2022), served themselves 360-degree VR videos that included various nature scenes (e.g., sunny forest, dense forest, shore of a rocky lake, floating icebergs, and sunny beach) while sitting in swivel chairs or lying in bed.

Moyle et al. (2018) showed participants images of a forest environment that include a river, accompanied by a background soundtrack incorporating peaceful, forest noises such as bird calls. Brimelow et al. (2020), used 360-degree videos contained in the virtual library consisting of various relaxing scenes, formed by various natural environments (e.g., forestal, aquatics, scenarios with animals, adventure, and travel).

### Quality assessment checklist

**Figure 2:**
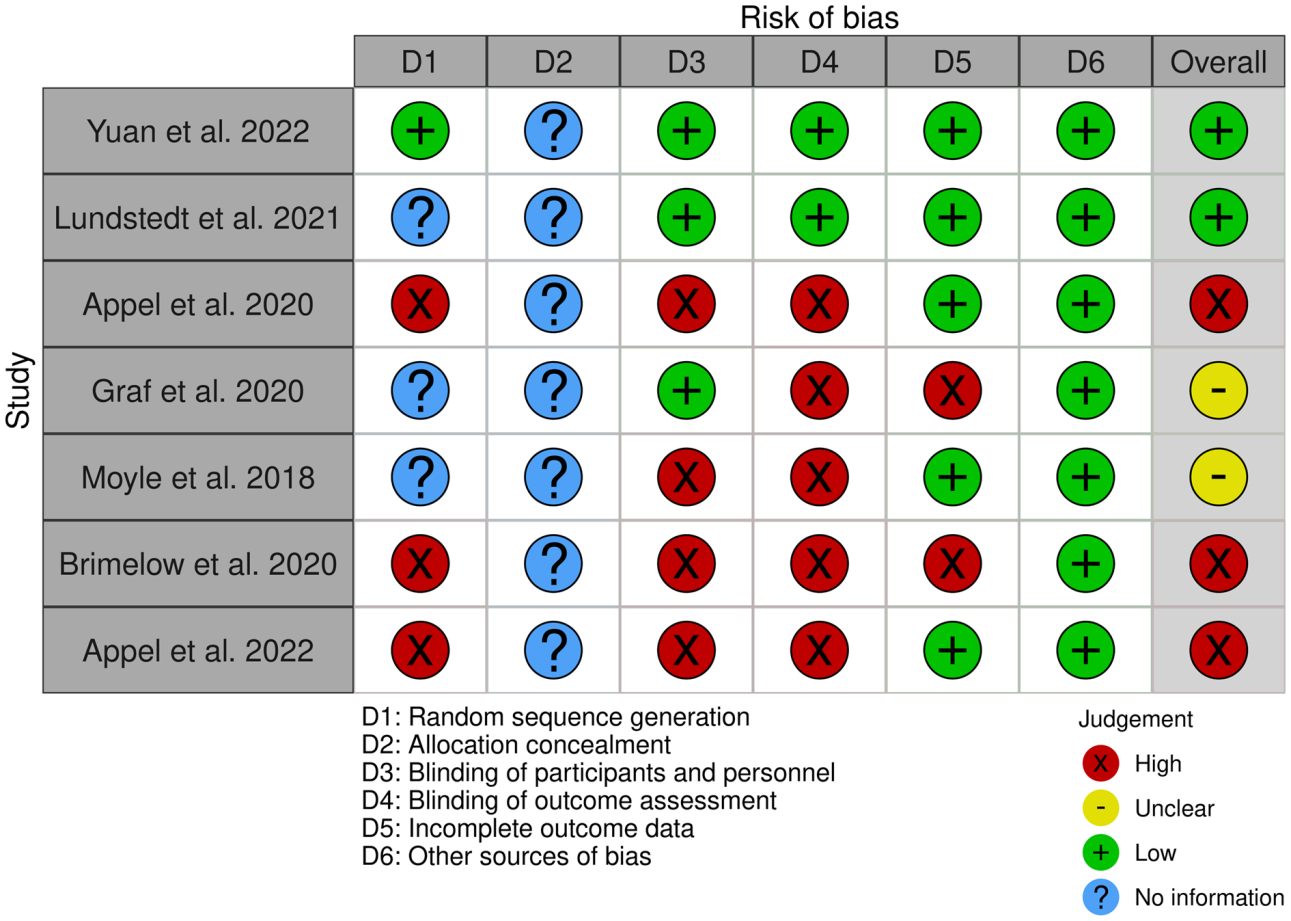
Risk of bias graph based on the Cochrane risk of bias tool (McGuinness and Higgins, 2021).

**Figure 3:**
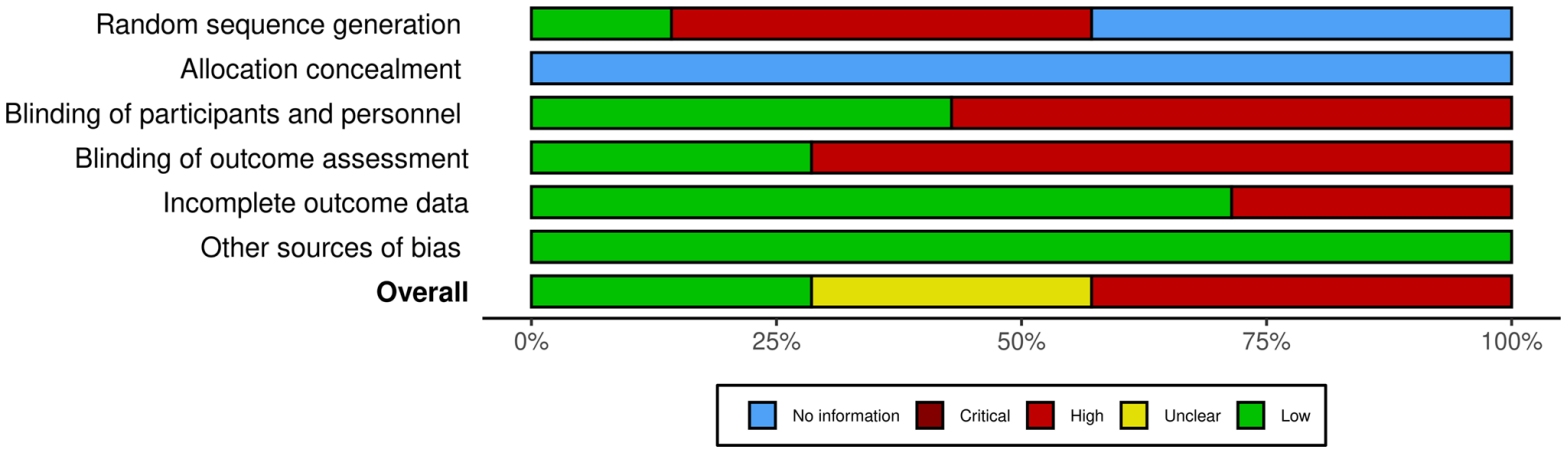
Risk of bias summary based on the Cochrane risk of bias tool (Higgins et al., 2022).

To determine the risk of bias for the selected papers, it has opted to follow the Cochrane handbook for systematic reviews of interventions (Lundh and Gøtzsche, 2008; McGuinness and Higgins, 2021; Higgins et al., 2022), using the results of this evaluation as presented in Figure 2. Two studies of our systematic review present a low risk of bias overall (Lundstedt et al., 2021; Yuan et al., 2022), three present a high risk of bias (Appel et al., 2020; Brimelow et al., 2020; Appel et al., 2022) and two present an unclear risk of bias (Moyle et al., 2018; Graf et al., 2020). All the papers present no sufficient information for the “allocation concealment” (D2) and low risk of bias for the “other sources of bias” (D6) shown in Figures 2, 3.

## Results

### Summary of findings

Yuan et al. (2022) conducted a field experiment to test the restorative effects of VR-simulated forest environment experiences on older people. They were based on the Attention Restoration Theory (ART) (Kaplan and Kaplan, 1989) and Stress Recovery Theory (SRT) (Ulrich et al., 1991). The authors investigated the different effects of a VR forest environment experience on psychological and physiological improvement in the investigated sample, assessing whether these effects were related to the participant’s different personalities. Finally, they sought to assess whether VR forestry experiences can increase the intention to engage *in vivo* forestry therapy. The study results showed that a brief experience in a VR forest generates immediate psychological restorative effects in older individuals, such as decreasing negative affect, increasing positive affect and enhancing stress recovery. Despite the initial record of significant psychological improvements at post-test compared with baseline, these effects were not maintained over time unless adequately reinforced. This study has shown that experiences in VR forest environments have greater effects on introverted individuals. Moreover, the study showed that participants had a greater intention to engage in real forest therapy.

Lundstedt et al. (2021) carried out a similar investigation in their study. Data were collected through observation of behavior and verbal expressions during the experiment. Participants showed their liking toward the aesthetic qualities of the environments through VR, as well as a fascination with the capabilities of technology. Negative reactions were recorded, such as anger caused by the desire to go outside and breathe fresh air. Participants showed much interest in virtual reality and the latter was able to promote social interactions among participants. The authors have found most expressions of aesthetic pleasure in relation to VR Island and participants express joy, especially in association theBlue. Residents expressed a fascination for the VR technology (especially in theBlue), the experience of the natural environment, its content and/or the strong feeling of presence. Residents experience mild discomfort when accidentally driving through or too close to objects, or when the water in VR Island appears cold and stormy. Most general positive reactions were expressed in relation to theBlue. Unlike the other studios, in this case the forest setting is contingent on the condition of the VR Island, which, however, contains various settings.

Appel et al. (2020), investigated the possibility of using virtual reality as a therapeutic tool for the older people with impaired sensory, motor, and/or cognitive ability. The results show that the participants recorded increases in calmness, happiness, energy and sense of relaxation following the VR experience. A significant increase was also recorded in feelings of confidence toward VR. Participants reported less anxiety, worry and stress. Negative emotional states included an increase in feelings of fatigue and loneliness following the implementation of the intervention. No side effects resulting from VR exposure, such as nausea or disorientation were recorded; participants were also able to physically explore virtual environments through head and body movements. This study, therefore, validates the hypothesis that immersive VR exposure is a feasible and safe approach to providing beneficial experiences for the older people with motor, sensory or cognitive disabilities.

Similar results were also achieved by the work of Graf et al. (2020). The authors subjected a sample of older people to an intervention implemented through VR, consisting of a virtual forest walk (VR Forest Walking) during which participants had to solve cognitive tasks. The conclusions show that the intervention with VR produced a change in positive affect and negative affect scores, but was not significant as the authors report [e.g., -positive affect: *t*(13) = −1.6, *p* = 0.134, *d* = −0.55; negative affect *t*(13) = 1.37, *p* = 0.195, *d* = −0.22], the medium-sized effect, according the authors, could indicate that this is due to the small sample size (normality has been ensured using the Kolmogorov–Smirnov test). Participants’ interest in technology was assessed; participants used the technology naturally and showed a desire to use it again. The participant’s expressions indicated a strong sense of presence that made them feel positive emotions: the participants interacted with the dog and cared for it. The authors have found high levels of flow from participants, but they were not significant. Virtual reality was found to be effective in stimulating the participants’ memory by evoking in them, through images of the forest, joyful memories of their past. The authors therefore found VR to be effective in treating older participants by showing the possibility of expanding the scenarios used during the intervention.

The study by Moyle et al. (2018) aimed to evaluate the effectiveness of a VR intervention of a forest environment on participants’ engagement, apathy and mood states. The results showed that during immersion in the VR forest, participants experienced higher levels of alertness and a higher degree of pleasure. However, the data also showed, at the group level, a greater increase in levels of fear/anxiety during the forest experience compared to the normative sample in an activity setting. The result is, however, influenced by the sample size, which also according to the authors is too small. The authors recorded reductions in apathy levels during the implementation of the experimental condition. However, apathy levels normalized shortly after the intervention ended. This may have induced a possible confounding effect, failing to define whether the reduced levels of apathy during the experience were related to the experience itself or the interaction with the staff.

The article by Brimelow et al. (2020) represents the first contribution to the literature about the feasibility of a VR intervention on a sample of people with varying degrees of cognitive impairment to reduce behavioral and psychological symptoms. Implementation of VR intervention significantly reduced depression from the pre-test to the post-test condition. The intervention was effective in improving the participants’ mood during the sessions. Positive effects were inconsistent across sessions; this could be caused, first, by the habituation that can result from prolonged exposure to VR. Participants showed a good tolerability of the VR intervention.

In their article, Appel et al. (2022) focused on a group of dementia patients admitted to a long-term care center who exhibit reactive behaviors, including those related to physical and emotional pain experience. The authors evaluated the feasibility of VR therapy (consisting of a collection of short 360° VR videos including various nature scenes). These videos were designed for persons with dementia, and the resulting benefits in order to reduce reactive behaviors, including those related to physical and emotional pain. VR therapy was examined both during specific targeted sessions in conjunction with events known to trigger reactive behaviors and during moments of free time. In the targeted sessions, the therapy was effective in reducing reactive behaviors triggered by the environment. The results identified VR therapy as acceptable and enjoyable for patients, showing potential effectiveness in reducing reactive behaviors, assisting in pain management and supporting enjoyment, relaxation and reminiscence.

With reference to five of the articles analyzed (Appel et al., 2020, 2022; Brimelow et al., 2020; Graf et al., 2020; Lundstedt et al., 2021), a comparison between different natural virtual scenarios was not made, but the virtual scenarios employed were used as a whole. Therefore, the results obtained in these studies do not give us specific indications regarding the effectiveness of forest therapy in VR compared to the other natural conditions tested. Rather, they demonstrate the effectiveness of the VR medium implemented with natural environments, but do not allow us to assume the specific benefits of forest therapy in VR.

## Discussion

As evidenced by research, life expectancies continue to increase (Mathers et al., 2015), leading to an increase in the number of older people, whose conditions, however, do not always allow them to be independent, resulting in high health and social costs (Kehusmaa et al., 2012). The living conditions of the older people have been the subject of study and attention (e.g., Hellström and Hallberg, 2001; Doblhammer and Ziegler, 2006; Parker and Thorslund, 2007). Forest therapy is an effective therapy *in vivo*, but little is known about its effectiveness in VR, although it may be beneficial especially for those who cannot go into nature due to their conditions. Therefore, this study sought to investigate evidence in the scientific literature concerning the beneficial effects of VR forest therapy and the forest environment in older people through a systematic review process. Findings confirm the hypothesis of a beneficial effect of forest therapy and forest environments through VR. Such types of studies, still show shortcomings regarding an older and bedridden sample. Aging naturally causes a reduction in the ability to engage in physical activity, especially when aggravated by the presence of one or more disease conditions. According to the results of the analyzed studies, the use of forest therapy with VR would go a long way in decreasing some psycho-physical effects typical of aging and bedridden conditions (e.g., improved mood and reduced stress). The increased length of time these individuals live with the medical condition results in demand for increased health care. Improving the health condition of the older people through this type of intervention can help reduce social costs. Moreover, given the stress-reducing effects of these interventions, they can also be used on the caregivers themselves. The systematic review reveals the need to compare different environments to see which one is more effective in delivering the effects because in many of the studies identified what emerges is only the efficacy of VR *per se* but is not clear which scenarios are more effective than others, because in some of the studies considered, environments with forest characteristics were presented together with other natural environments, and thus it is not possible to verify whether the results obtained were attributable to forest environments. In students and adults’ samples, however, differences were found depending on the natural environment used in VR (e.g., Theodorou et al., 2023a, b).

It is worth mentioning that VR has also been employed with older people using virtual scenarios other than forest ones, such as beaches, coasts, and underwater, or a distant view of mountains (e.g., Reynolds et al., 2018; Orr et al., 2021). Nonetheless, although VR has been mainly used with older people for therapeutic and rehabilitation purposes (e.g., Rendon et al., 2012; Lee, 2017; Kamińska et al., 2018; Tsao et al., 2019), studies to date that have investigated its use focusing on the beneficial effects of virtual natural environments *per se* are still scarce. Therefore, due to the different modes in which VR can be employed in older people, it would also be appropriate to understand in future research what the most effective use of this tool on this specific target population could be in order to minimize risks and maximize its beneficial effects.

The present study highlights the need for further research and especially for their standardization. Specifically, the highlighted studies showed heterogeneity in tools used. This heterogeneity can also be found in the type of stimulation (video-derived or software-created forest environment) and instrumentation used to reproduce the stimulation. Standardization would allow us to adopt the best device possible to achieves better results in improving older people’s psycho-physical well-being.

### Limitations, strengths, and future perspectives

The study is not without limitations. The first one concerns the low number of studies using forest-related technology with older people samples. In addition, many of these studies have a numerically small sample size. Nonetheless, the difficulty of obtaining high samples for this age group should be considered, due in part to the necessary screening (unimpaired vision and hearing, etc.). These limitations highlight the need for further studies, with larger samples. A general limitation of these studies may also be the heterogeneity of the type of instrumentation used and the type of forest setting and outcomes analyzed. In this regard the use of PSDs (perceived sensory dimensions) could be of help in order to standardize forest images and videos (Grahn and Stigsdotter, 2010). Other limitation pertains to the lack of verification of long-term effects. Future studies to investigate more thoroughly the temporal impact of such interventions are needed. Another limitation consists of publication bias of positive results The strengths of this systematic review are that, at present, this is the first systematic review investigating state of the art concerning the use of forest environments through VR on an older population. A further strength lies in the fact that this systematic review highlights the need to study the differences between different types of natural environments in a sample of older people in greater depth. Our work has shown that for this type of sample, studies using forest therapy through VR are scarce, and some of them do not only use forest therapy, but several natural environments together, making it impossible to understand the real benefits of the specific natural environment. Given the specific population, future studies should also attend to the impact of medications taken by patients, and specific psycho-physical conditions. Regarding bias, only two articles were identified by the authors of this systematic review as having a low risk of bias. This finding highlights the need for more methodologically sound studies.

Finally, the results of this systematic review shed light on the relevance of further investigating the topic by also publishing and disseminating those studies whose results are negative or non-significant. Focusing only on positive and significant results might increase the risk of publication bias (Dickersin and Min, 1993), resulting in the skewing of overall conclusions by overestimating positive effects and, at the same time, underestimating the negative ones (Dwan et al., 2008; Mueller et al., 2013). Although a careful examination of the gray literature was conducted, no further eligible studies emerged through the keywords adopted. Nonetheless, especially with this target population, addressing studies’ negative and/or non-significant results with the VR medium would allow the set of increasingly safe and precise interventions, providing more robust benefits for their health and well-being.

## Conclusion

The studies analyzed in this systematic review highlight the usefulness of this type of intervention combining forest setting and VR, where the latter serves as a medium for transmitting the beneficial effects of the former. Although not all studies show significant effects in all outcomes, the effects found would seem sufficiently encouraging. The use of natural environments, and those of forest nature more specifically, contribute to improving psycho-physical well-being. However, due to the *in vivo* nature of such treatments, this type of intervention is not accessible to all types of patients. The contribution of virtual reality in this regard, given its immersive experience, makes up for this gap and allows exposure to natural environments even for people who are bedridden or have motor difficulties, especially the older people. This systematic review highlighted the need to design further studies having a more structured, rigorous and standardized methodology. More specifically, future studies should recruit well-powered samples including a control group and testing long term effects of exposure to nature through VR. The effects of this type of intervention would have not only benefits on direct recipients but also on indirect recipients (caregivers, nurses, etc.), leading to a reduction in social costs.

## Data availability statement

The original contributions presented in the study are included in the article/supplementary material, further inquiries can be directed to the corresponding author.

## Author contributions

DC: Conceptualization, Investigation, Methodology, Writing – original draft, Writing – review & editing. LR: Writing – original draft, Writing – review & editing. EZ: Investigation, Writing – original draft. GC: Writing – review & editing. AP: Supervision, Writing - review & editing.

## Footnotes

^1^ https://prisma-statement.org/
